# Safety and efficacy of glibenclamide on functional outcomes in ischemic and hemorrhagic stroke: a systematic review and meta-analysis of randomized clinical trials

**DOI:** 10.3389/fneur.2025.1609101

**Published:** 2025-06-27

**Authors:** Ola Bin Shilash, Hussam Alhathlol, Rana Alduhaysh, Razan Almufarriji, Mohammed Bafaquh

**Affiliations:** ^1^College of Medicine, King Saud bin Abdulaziz University for Health Sciences, Riyadh, Saudi Arabia; ^2^King Abdullah International Medical Research Center, Riyadh, Saudi Arabia; ^3^Department of Neurosurgery, National Neuroscience Institute, King Fahad Medical City, Riyadh, Saudi Arabia; ^4^Department of Neuro-Oncology, King Faisal Specialist Hospital and Research Center, Riyadh, Saudi Arabia

**Keywords:** glibenclamide, stroke, ischemia, SAH, hemorrhage

## Abstract

**Background:**

Secondary brain injuries, including delayed cerebral ischemia, neuroinflammation, and stroke induced cerebral edema can occur following both ischemic and hemorrhagic strokes, contributing to a negative impact on clinical outcomes. Glibenclamide, a sulfonylurea antidiabetic medication, has shown potential in minimizing these consequences by targeting the SUR1-TRPM4 channel. However, glibenclamide’s therapeutic effectiveness and safety in stroke patients remain unknown. Therefore, this systematic review aims to assess the safety and efficacy of glibenclamide in improving outcomes following both ischemic and hemorrhagic strokes.

**Methods:**

Four databases were searched for RCTs published up to November 2024. Studies were included if they involved adult patients with ischemic stroke, hemorrhagic stroke, or subarachnoid hemorrhage, and reported relevant safety and efficacy outcomes. Efficacy outcomes were measured using the Modified Rankin Scale at 3 and 6 months. Safety outcomes included adverse events such as hypoglycemia, hydrocephalus, and mortality.

**Results:**

Data from six RCTs, involving 555 patients (280 intervention, 275 control), were included: 4 trials in subarachnoid hemorrhage, one trial in ischemic stroke, and one in hemorrhagic stroke. At 3 months, the pooled odds ratio (OR) for poor functional outcomes was 0.98 (95% CI: 0.65–1.48), and at 6 months, 0.52 (95% CI: 0.24–1.12; *p* = 0.094), with no significant differences between glibenclamide and placebo. Safety analysis showed a significant increase in symptomatic hypoglycemia (OR 4.69, 95% CI: 1.45–15.23; *p* = 0.010) but no significant differences for hydrocephalus (OR 1.60, 95% CI: 0.76–3.37; *p* = 0.220) or mortality (OR 0.57, 95% CI: 0.32–1.05; *p* = 0.071). Delayed cerebral ischemia (DCI) showed a borderline reduction in risk (OR 0.43, 95% CI: 0.18–1.00; *p* = 0.051) in the treatment group.

**Conclusion:**

In patients with ischemic or hemorrhagic stroke, glibenclamide demonstrates a favorable safety profile but shows limited efficacy in improving functional outcomes. The elevated risk of hypoglycemia emphasizes the necessity of using this medication with caution.

## Introduction

Stroke remains one of the leading causes of morbidity and mortality globally, with an increasing burden due to rising incidents and prevalent cases over the past three decades (1). It is broadly categorized into ischemic stroke, caused by vascular occlusion, and hemorrhagic stroke, which includes intracerebral hemorrhage (ICH) (2). Despite advancements in acute stroke care, such as improved diagnostic tools and interventions, long-term outcomes remain suboptimal due to secondary complications like neuroinflammation, cerebral edema, and delayed neuronal injury (1, 2). This highlights the urgent need for novel therapeutic approaches targeting these mechanisms. Current treatments primarily concentrate on managing the acute phase, including reperfusion intervention (1, 2). Nevertheless, there is a considerable deficiency in therapies aimed at the molecular mechanisms that contribute to secondary injury (3). Glibenclamide, a well-known sulfonylurea antidiabetic medication, is one promising therapeutic approach. Glibenclamide works by blocking the sulfonylurea receptor 1 (SUR1)—transient receptor potential melastatin 4 (TRPM4) channel complex, which is essential in the pathophysiology of many central nervous system (CNS) injuries, including aSAH (4). The activation of the SUR1-TRPM4 channel has been linked to vasogenic edema, neuroinflammation aggravation, and neuronal integrity impairment. Glibenclamide, which targets this channel, has the ability to minimize cerebral edema, limit neuronal death, and reduce inflammation, therefore enhancing neurological recovery (5). Preclinical research has provided solid evidence for glibenclamide’s neuroprotective properties (6). In animal models of ischemic brain damage, glibenclamide treatment has been demonstrated to decrease vasogenic edema, reduce infarct volume, and enhance functional recovery (6). Building on this basis, preliminary clinical studies have assessed the function of glibenclamide in the setting of stroke. For example, recent research found that high-dose oral glibenclamide significantly reduced radiological indicators of cerebral edema during 10 days of therapy, implying possible advantages in preventing decompressive surgeries (7). Other trials, however, have shown conflicting results, with some failing to detect substantial increases in functional outcomes or decrease in mortality rates (3). While preliminary data suggests that it can minimize vasospasm and enhance perfusion, inconsistencies in research design, dosage regimens, and outcome measures have restricted the generalizability of findings (5). Furthermore, some concerns do exist about glibenclamide’s safety profile, including its possible impact on glucose homeostasis and other systemic side effects in non-diabetics (4, 8, 9).

To overcome these gaps, this systematic review and meta-analysis will analyze data from randomized controlled trials to assess the effectiveness and safety of glibenclamide in stroke. This study aims to clarify the influence of glibenclamide on major clinical outcomes.

## Methods

### Search strategies

Following the Preferred Reporting Items for Systematic Reviews and Meta-Analyses (PRISMA) guidelines, four online databases were used to search relevant literature: PubMed/Medline, Cochrane, Science Direct, and Web of Science. We only included peer-reviewed journals published from inception until November 2024 published in English only. The keywords used in this study are (Glibenclamide OR Glyburide) AND (Aneurysmal subarachnoid hemorrhage OR SAH OR Intracranial aneurysm OR Ischemic stroke OR Hemorrhagic stroke).

### Screening and selection of studies

All studies were retrieved through database searches and entered on an Excel spreadsheet. Initially, two researchers independently reviewed the titles and abstracts. Any disagreements were resolved by conducting a full-text review of the article. Next, the full texts of the selected studies were assessed for eligibility by two researchers working independently. Data from the full-text articles were extracted using a standardized form, with two independent researchers performing the extraction.

### Study design and criteria

#### Inclusion

Studies evaluating adults with stroke (confirmed by clinical assessment and imaging).

Only RCTs reporting outcomes relevant to the research question will be included.

#### Exclusion

Studies that include adolescents (under 18 years of age) and elderly people (over 74).

Studies that include patients currently using glibenclamide.

Studies written in any language other than English.

There will be no restrictions on the geographical location of studies.

**Figure 1:**
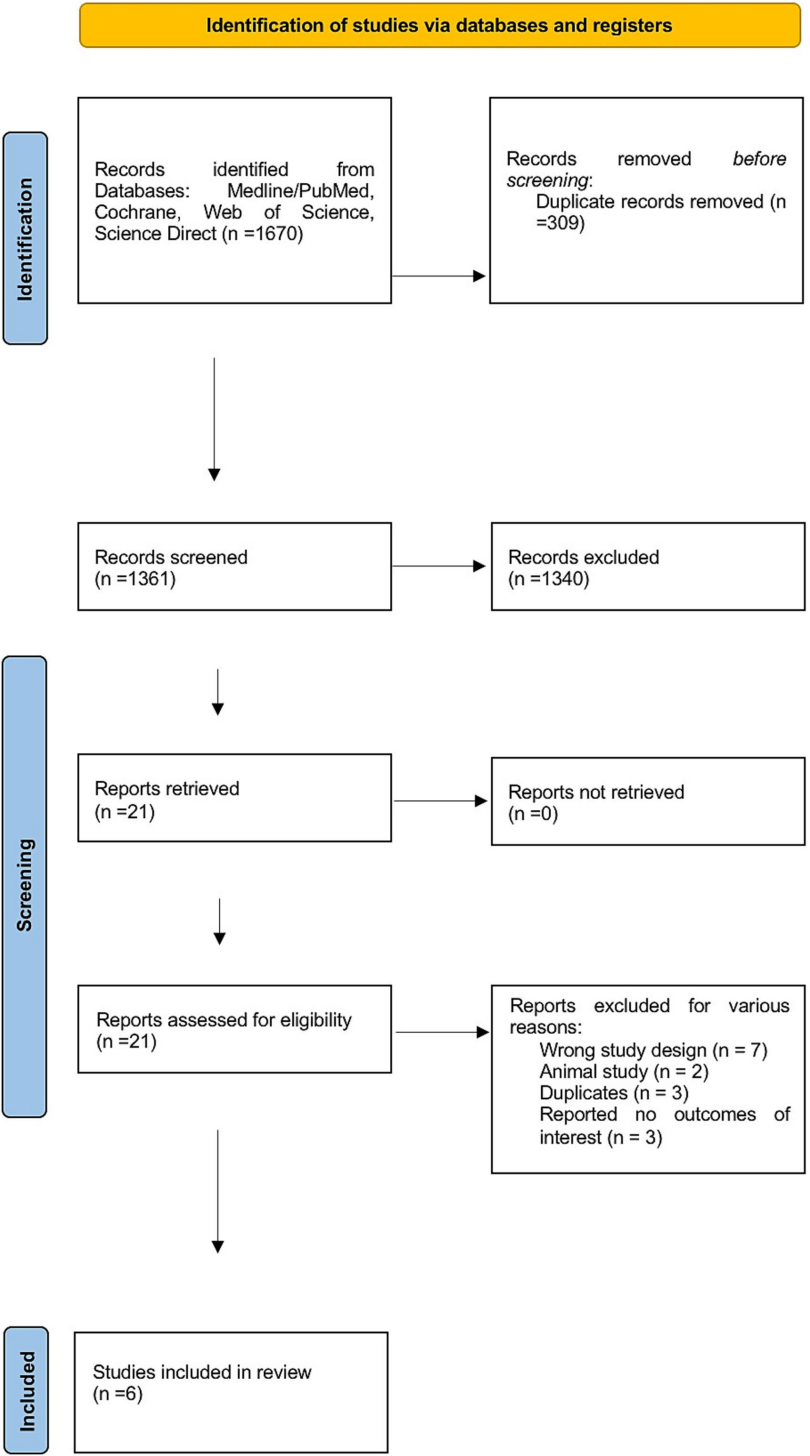
PRISMA flowchart for the screening process.

The flowchart illustrates the systematic review process of our study, starting with the identification of 1,670 initial records. After removing duplicates, 1,361 studies remained. These studies were carefully reviewed by title and abstract to assess their eligibility for inclusion. A total of 21 studies were selected for full-text screening, resulting in the exclusion of 15 studies for various reasons illustrated in Figure 1. Ultimately, six studies were included in the final analysis.

### Risk assessment

The risk of bias for included studies was assessed using the Cochrane Risk of Bias Tool for randomized controlled trials (RCTs). The tool assesses selection, performance, detection and attrition risk of bias. Risk of bias was assessed independently by two reviewers to ensure consistency and reliability. Any disagreements between reviewers were resolved through discussion.

### Statistical analysis

Records collected as median and interquartile ranges (IQR) were converted to their respective mean and standard deviation values as described previously (10). The efficacy outcomes in this study were defined as poor functional outcomes, measured by the Modified Rankin Scale (mRS 3–5) at 3 and 6 months. Safety outcomes included reported adverse events (hydrocephalus, hypoglycemia, or cerebral infarction events) and death. Meta-analyses were conducted using the Mantel–Haenszel method to calculate pooled odds ratios (OR) with corresponding 95% confidence intervals. Subgroup analyses were performed based on the type of stroke. Heterogeneity across studies was assessed using the *I*^2^ statistic to quantify the percentage of total variation due to heterogeneity. Forest plots were generated to illustrate pooled outcomes, subgroup analyses, and the results of sensitivity analyses, which assessed the effect of excluding each study sequentially. Statistical analyses were conducted using RStudio (version 2024.9.1.394, Boston, MA, United States) with R version 4.4.2. A *p*-value <0.05 was considered statistically significant.

## Results

### Characteristics of studies and patients

A total of six RCTs were analyzed in the current study. Trials were published between 2018 and 2024. Three trials were conducted in China (4, 7, 9), two trials in Brazil (8, 11) and one trial in the United States (12). A total of 555 patients were recruited across the included studies, of whom 280 (50.5%) and 275 (49.5%) patients were allocated to the intervention and control groups, respectively. Gender distribution was available in five studies, where 241 males and 249 females were included out of 490 patients, accounting for 49.2 and 50.8%, respectively. One trial included patients with ischemic stroke (12), one trial included those with hemorrhagic stroke (9), while the remaining trials included patients with aneurysmal subarachnoid hemorrhage (aSAH). More details about patients are included in Table 1.

**Table 1:** Characteristics of the included trials and patients.

Authors	Country	Sample size	Gender	Study groups	Type of stroke	Location of stroke/aneurysm		Stroke management	Baseline NIHSS score, median (IQR)
			M/F	I/C		Anterior circulation	Posterior circulation		
da Costa et al. (8)	Brazil	78	19/59	38/40	aSAH	NR	NR	Surgical clipping: Control *n* = 30, Intervention *n* = 20 Endovascular coiling: Control *n* = 10, Intervention *n* = 18	NR
Feng et al. (7)	China	56	29/27	28/28	aSAH	Intervention: 23 Control: 25	Intervention: 5 Control: 3	Vascular embolisation intervention: Intervention *n* = 26, Control *n* = 24	NR
Lin et al. (4)	China	111	53/58	57/54	aSAH	Intervention: 2 Control: 4	Intervention: 1 Control: 0	NR	NR
Sheth et al. (12)	US	65	NR/NR	35/30	Ischemic stroke	65	0	IV rtPA: (61%)	Intervention: 19 (16–22), Control 21: (16–23)
Windlin et al. (11)	Brazil	45	12/33	23/22	aSAH	NR	NR	Microsurgery: *n* = 27 Embolization: *n* = 18	NR
Zhao et al. (9)	China	200	128/72	99/101	Hemorrhagic stroke	200	0	Standard care (BP control)	Intervention: 7.0 (5.0–10.0), Control: 8.0 (4.0–12.0)

aSAH, aneurysmal subarachnoid hemorrhage; I, the intervention group; C, the control group; M, male; F, female; IQR, interquartile range.

### Characteristics of interventions and controls

The reported doses of glibenclamide ranged from 1.25 mg administered three times daily to 15 mg daily. The route of administration was primarily oral or via nasogastric tube (NGT), except for one trial (12), which used an intravenous (IV) regimen with a bolus dose followed by continuous infusion over 72 h. Treatment durations varied from 3 to 21 days across studies. Placebo types included starch, vitamin B1, and standard care, while two studies (11, 12) did not specify placebo details (Table 2).

**Table 2:** Characteristics of interventions and controls.

Author	Glibenclamide dose	Route of administration	Duration of treatment	Type of placebo
da Costa et al. (8)	5 mg	Oral, NGT	21 days	Starch
Feng et al. (7)	15 mg	Oral, NGT	10 days	Vitamin B1
Lin et al. (4)	3.75 mg	oral	7 days	None
Sheth et al. (12)	IV 0.13 mg bolus during first 2 min, followed by IV infusion at a rate of 0.16 mg/h for 6 h, followed by 0.11 mg/h for 66 h	IV	3 days	NR
Windlin et al. (11)	5 mg	Oral, NGT	21 days	Placebo (not otherwise defined)
Zhao et al. (9)	1.25 mg, 3 times/day	oral	7 days	Standard care (not otherwise defined)

### Results of the efficacy outcomes

**Figure 2:**
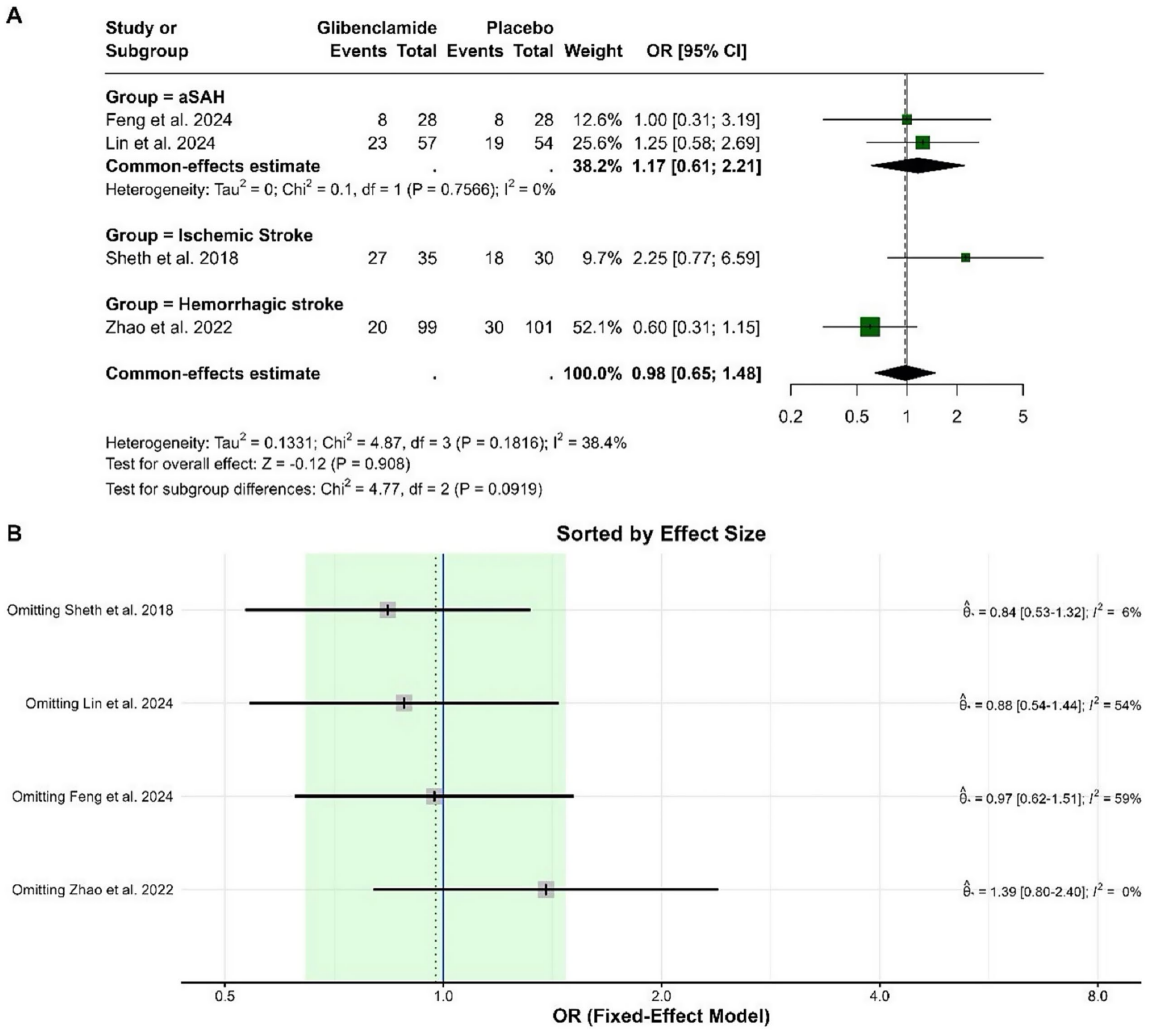
Forest plots depicting the effect sizes **(A)** and sensitivity analysis **(B)** for the meta-analysis of a poor functional outcome (mRS 3–5) at 3 months.

The pooled odds ratio (OR) for a poor functional outcome (mRS 3–5) at 3 months was 0.98 (95% CI: 0.65 to 1.48), indicating no statistically significant difference between glibenclamide and placebo. Heterogeneity was moderate, with an I-squared of 38.4%. Subgroup analysis by stroke type showed no statistically significant effects in any group. The pooled ORs were 1.17 (95% CI: 0.61 to 2.21) for aneurysmal subarachnoid hemorrhage, 2.25 (95% CI: 0.77 to 6.59) for ischemic stroke, and 0.60 (95% CI: 0.31 to 1.15) for hemorrhagic stroke. The test for subgroup differences was not statistically significant (chi-squared = 4.77, *p* = 0.092). Based on the sensitivity analysis, the omission of the Zhao et al. (9) study reduced heterogeneity to 0% and shifted the pooled effect size to an OR of 1.39 (95% CI: 0.80 to 2.40), indicating that this study contributed substantially to the observed heterogeneity and overall effect size (Figure 2).

**Figure 3:**
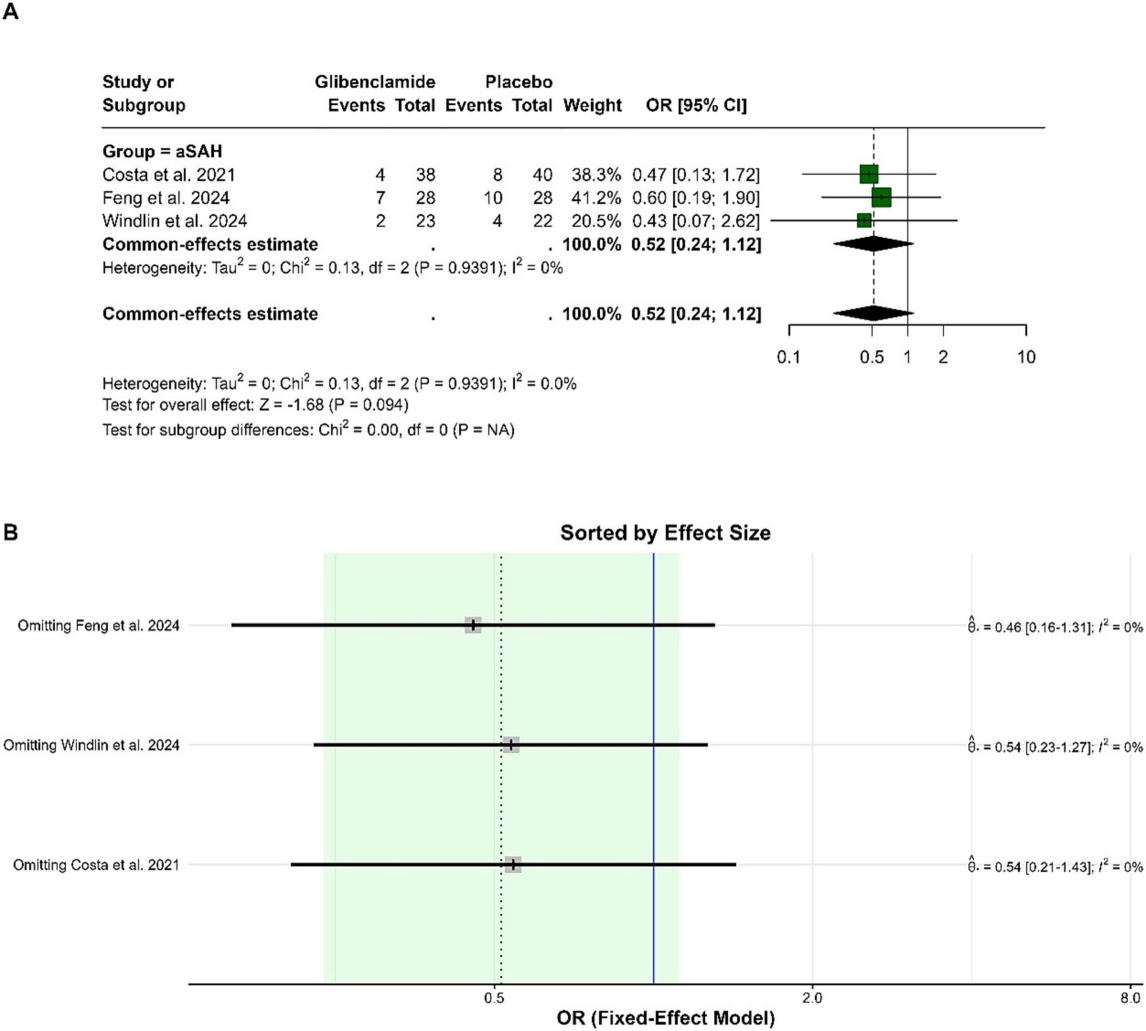
Forest plots depicting the effect sizes **(A)** and sensitivity analysis **(B)** for the meta-analysis of a poor functional outcome (mRS 3–5) at 6 months.

For the poor functional outcome (mRS 3–5) at 6 months, the pooled OR was 0.52 (95% CI: 0.24 to 1.12), indicating no statistically significant difference between glibenclamide and placebo (*p* = 0.094). There was no heterogeneity in the analysis, with I-squared at 0%. Subgroup analysis was not applicable as all studies focused on aneurysmal subarachnoid hemorrhage. Sensitivity analysis demonstrated consistent findings, with the pooled OR remaining similar when any single study was omitted (Figure 3).

### Results of safety outcomes

**Figure 4:**
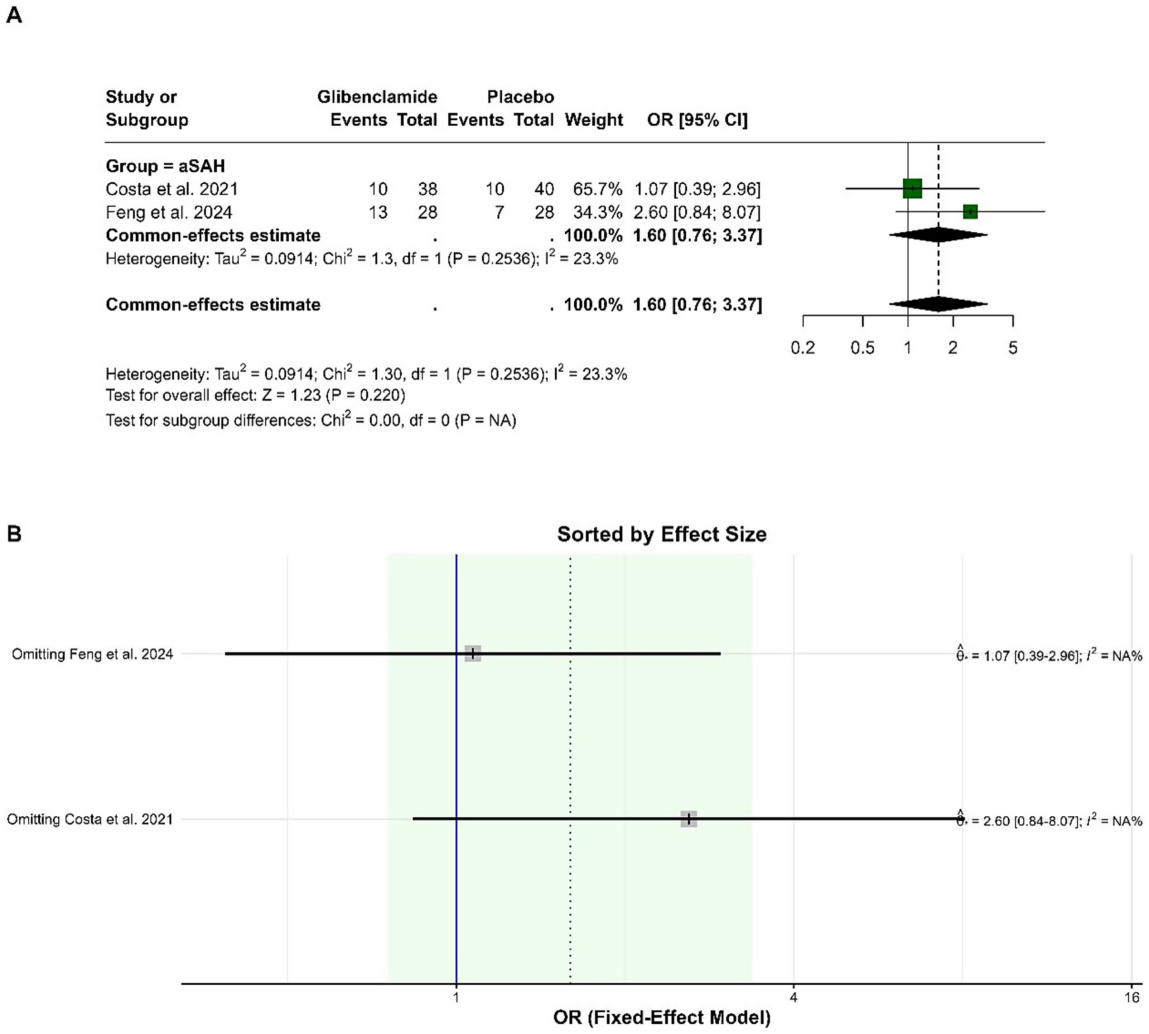
Forest plots depicting the effect sizes **(A)** and sensitivity analysis **(B)** for the meta-analysis of hydrocephalus.

The pooled OR for the occurrence of hydrocephalus was 1.60 (95% CI: 0.76 to 3.37), indicating no statistically significant difference between glibenclamide and placebo (*p* = 0.220). Heterogeneity was low, with an I-squared of 23.3%. Sensitivity analysis showed consistent results, with the pooled OR remaining within a similar range when either study was omitted (Figure 4).

**Figure 5:**
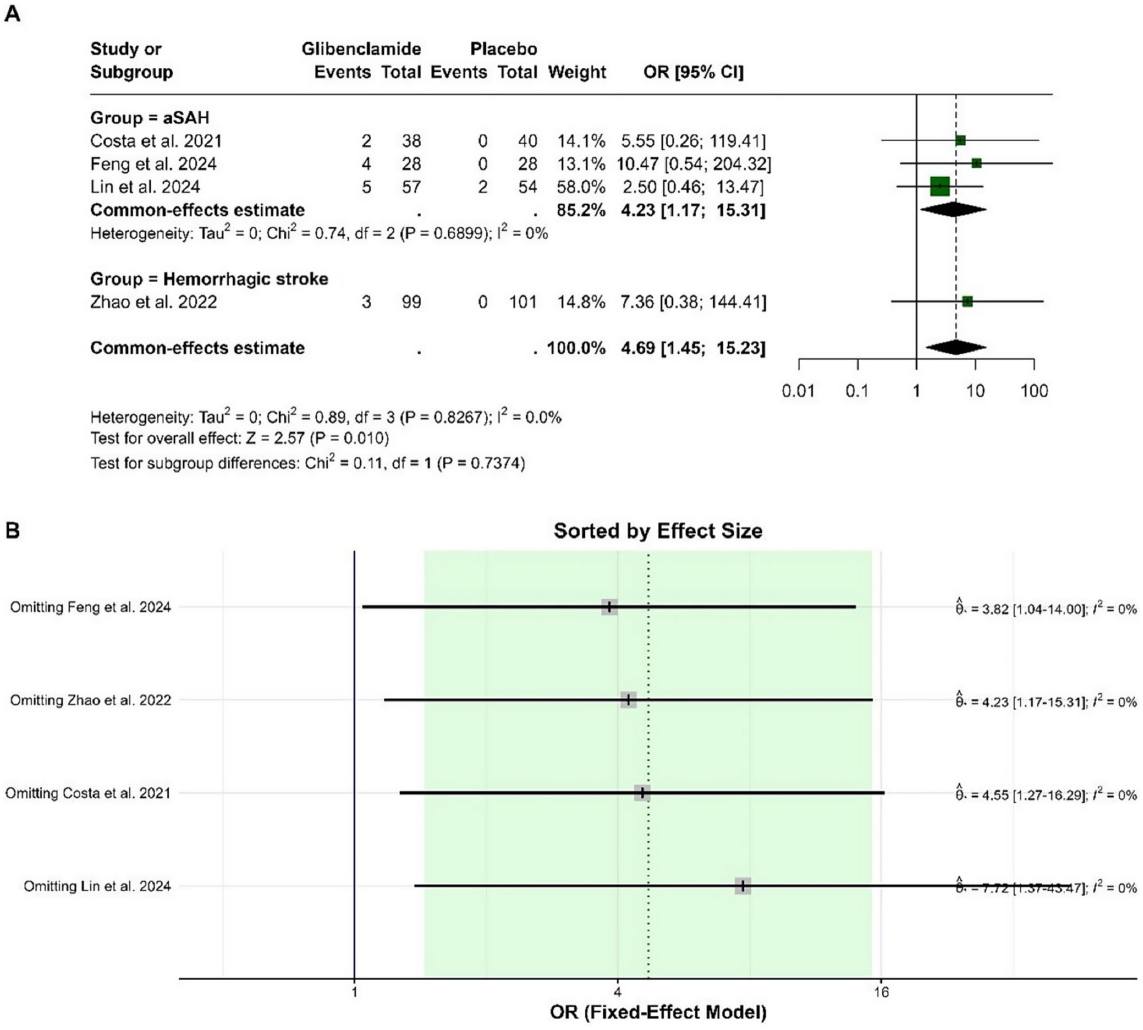
Forest plots depicting the effect sizes **(A)** and sensitivity analysis **(B)** for the meta-analysis of hypoglycemia.

The pooled OR for hypoglycemic events was 4.69 (95% CI: 1.45 to 15.23), indicating a statistically significant increase in the odds of hypoglycemia with glibenclamide compared to placebo (*p* = 0.010). Heterogeneity was negligible, with an I-squared of 0%. Sensitivity analysis showed that the pooled OR remained consistently significant when individual studies were omitted, with no impact on heterogeneity (Figure 5).

**Figure 6:**
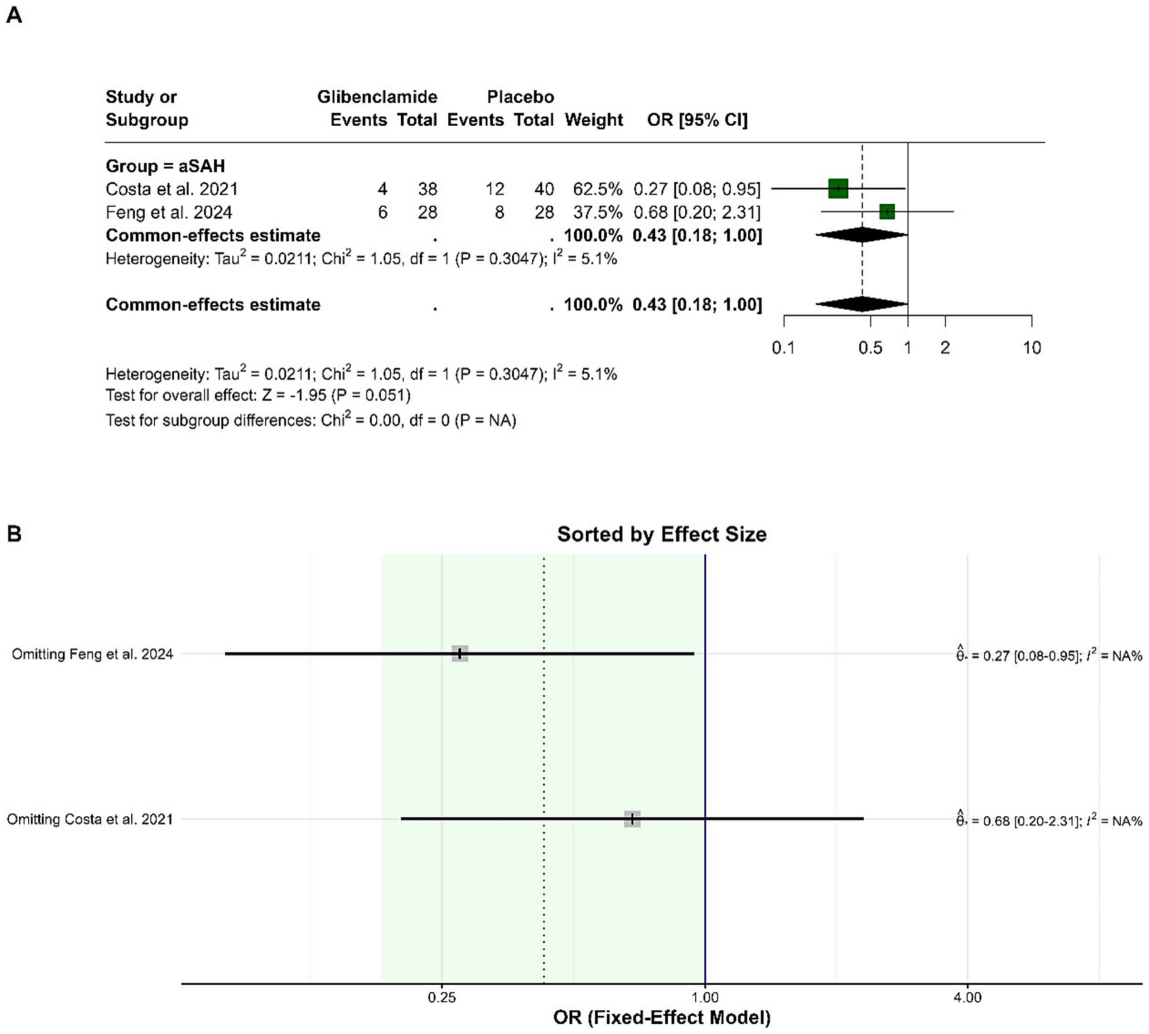
Forest plots depicting the effect sizes **(A)** and sensitivity analysis **(B)** for the meta-analysis of DCI.

The pooled OR for delayed cerebral ischemia (DCI) was 0.43 (95% CI: 0.18 to 1.00), with a borderline statistically significant reduction in risk for patients receiving glibenclamide compared to placebo (*p* = 0.051). Heterogeneity was low, with an I-squared of 5.1%. Sensitivity analysis showed that the pooled OR ranged from 0.27 to 0.68 when individual studies were omitted, and heterogeneity remained low (Figure 6).

**Figure 7:**
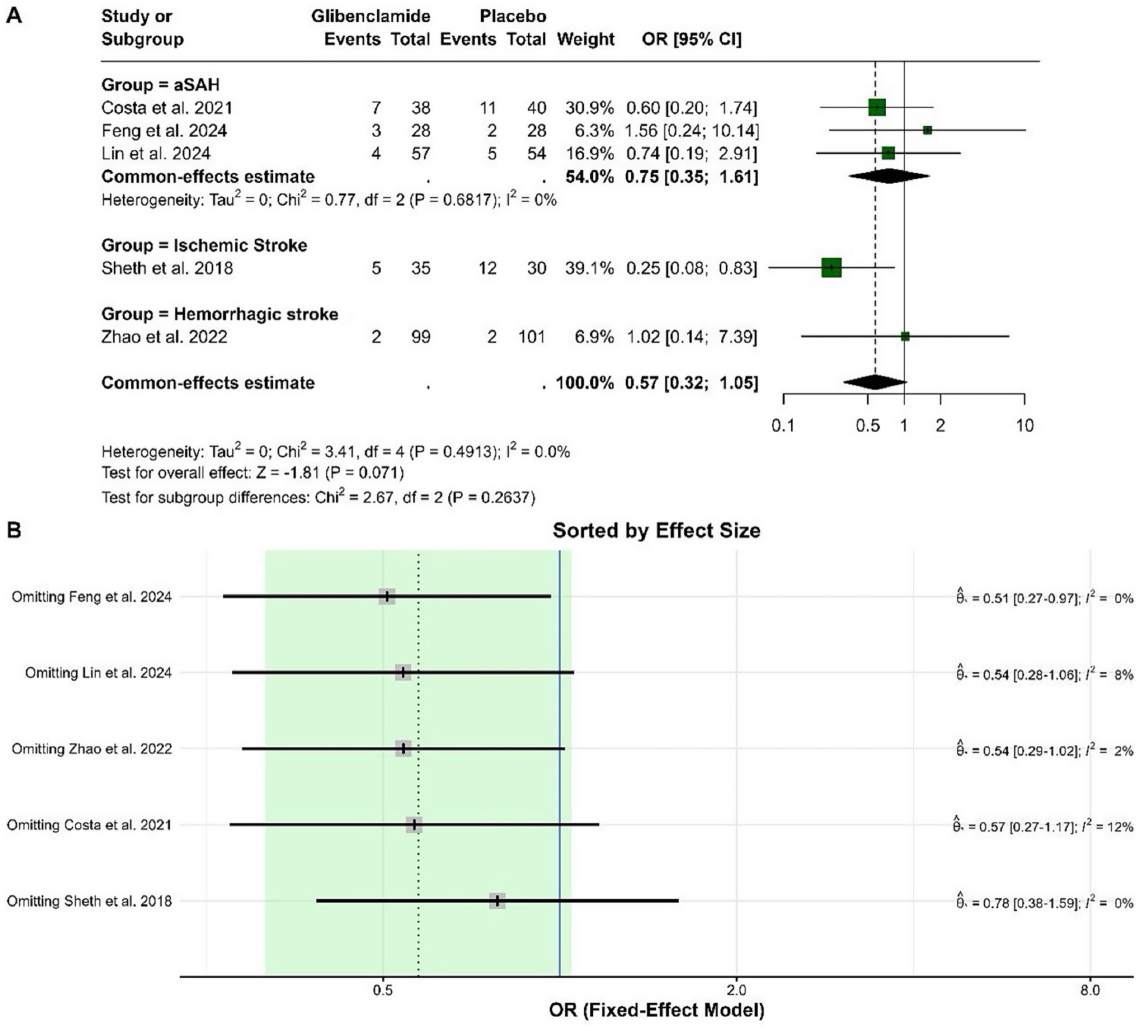
Forest plots depicting the effect sizes **(A)** and sensitivity analysis **(B)** for the meta-analysis of death.

The pooled OR for death was 0.57 (95% CI: 0.32 to 1.05), indicating no statistically significant difference between glibenclamide and placebo (*p* = 0.071). Heterogeneity was negligible, with an I-squared of 0%. Subgroup analysis did not show significant differences across stroke types, and the test for subgroup differences was not statistically significant (*p* = 0.264). Sensitivity analysis showed that the pooled OR remained stable and within a similar range, with heterogeneity remaining low regardless of the study omitted. These results indicate the robustness of the findings (Figure 7).

### Publication bias assessment

**Figure 8:**
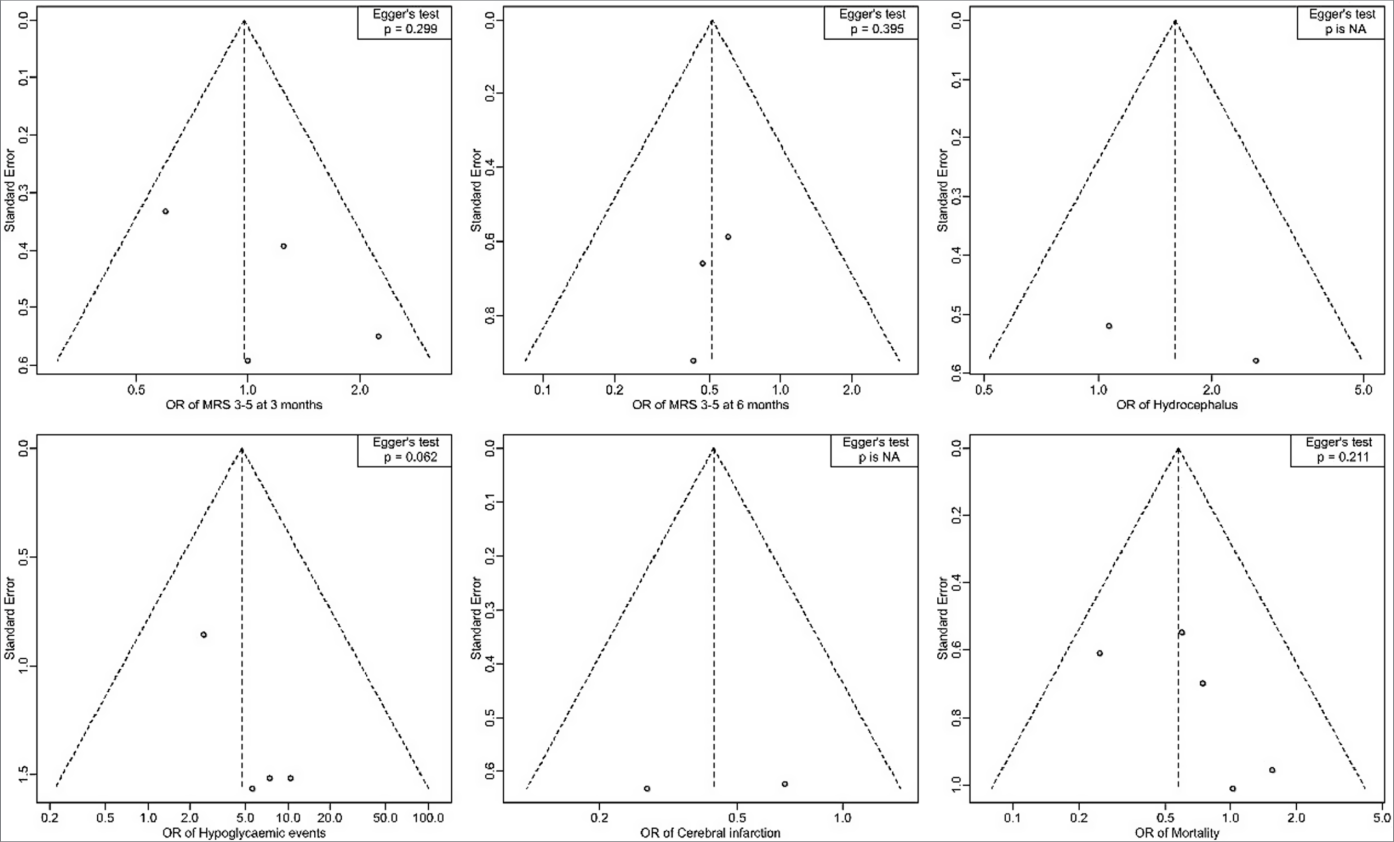
Forest plots for publication bias assessment by Egger’s test.

**Figure 9:**
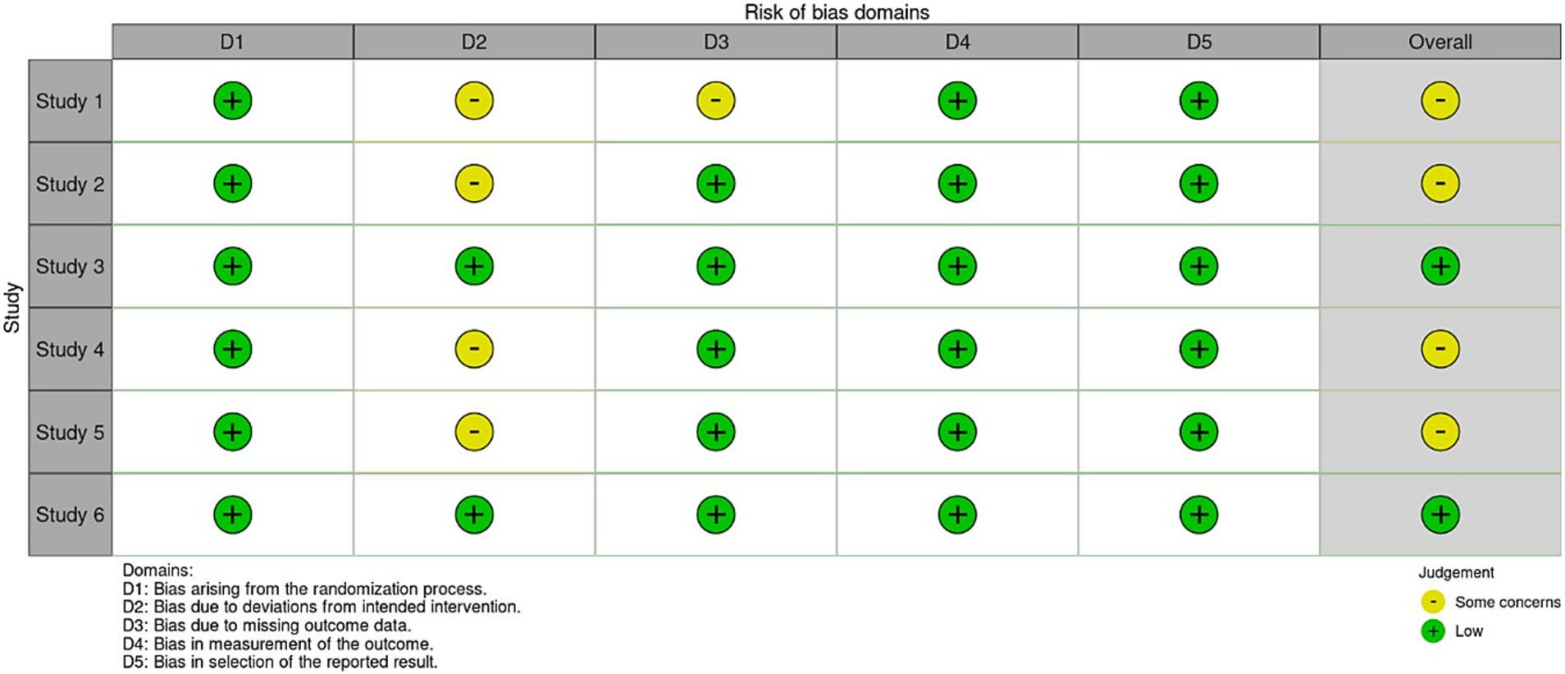
Risk of bias assessment by Cochrane tool.

The funnel plots for the outcomes suggested no significant publication bias, since effect sizes were evenly distributed around the main effect estimate. This observation was supported by Egger’s test results, which showed no statistical evidence of publication bias for mRS at 3 months (*p* = 0.299), mRS at 6 months (*p* = 0.395), hypoglycemic events (*p* = 0.062), and mortality (*p* = 0.211) (Figure 8). Publication bias assessment was not performed for hydrocephalus and cerebral infarction events because they were reported in two studies. Using the Cochrane risk of bias assessment tool, two studies had low risk of bias (4, 8), while the rest had some concerns (Figure 9).

## Discussion

Our findings revealed that glibenclamide did not achieve a statistically significant improvement in functional outcomes as measured by the modified Rankin Scale. According to the individual RCTs results, glibenclamide showed no significant improvements in mRS scores with only a single trial demonstrating a trend toward functional benefit at 90 days after adjustment for age (odds ratio, 2.31; *p* = 0.07) in glibenclamide group (12). A systematic review by dos Santos et al. (13) reported a notable reduction in poor functional outcomes (mRS ≥4) among patients with ischemic strokes, indicating potential benefits of glibenclamide. These findings highlight a disparity between aggregated and individual study results, raising questions about variability in patient populations, study designs, and intervention protocols. The underlying mechanisms of glibenclamide’s functional benefits may extend beyond infarct volume reduction, such as its impact on neurogenesis and neural repair, as supported by previous evidence (6). The present study found no significant impact of glibenclamide on hydrocephalus, as the current evidence of its effect on hydrocephalus remains limited and inconclusive. There is, however, well-established evidence demonstrating its efficacy in reducing malignant cerebral edema compared to placebo group (7, 9). These effects are attributed to glibenclamide’s inhibition of SUR1-TRPM4 channels, which play a critical role in preventing cytotoxic edema and ionic imbalances (14). Moreover, a visible decreasing trend in cases of delayed cerebral ischemia has been shown in the treatment group, with one trial reporting similar results between the two groups (4, 11). Hypoglycemia emerged as a significant concern in in this meta-analysis, aligning with another study highlighting frequent hypoglycemic episodes requiring intervention (13). This warrants further attention due to its potential to limit therapeutic application. Strategies such as careful dosing adjustments, timing of administration, and potential combination therapies with glucose-stabilizing agents may help optimize safety and efficacy (15).

### Limitations

Despite such results, several limitations must be acknowledged. Variability in treatment protocols, ranging from intravenous administration to continuous subcutaneous infusion, complicates direct comparisons. Additionally, small sample sizes limit the generalizability of findings. Furthermore, rigorous evaluation of glibenclamide long-term effects on cognitive outcomes, health-related quality of life, and comprehensive stroke recovery trajectories is imperative to establish its definitive role in clinical practice.

## Conclusion

In conclusion, this study evaluates the effectiveness and safety of glibenclamide in stroke management. While preliminary evidence from preclinical and early clinical studies suggests potential neuroprotective benefits, our analysis found no statistically significant improvement in functional recovery or mortality outcomes at 3 and 6 months. The safety profile of glibenclamide raised concerns regarding its potential to induce hypoglycemia, although the risk of hydrocephalus and cerebral infarction appeared comparable to placebo. Given the limited evidence on its clinical impact, further well-designed, large-scale randomized controlled trials are warranted to more definitively determine the role of glibenclamide as a therapeutic agent in stroke. The findings underscore the need for cautious interpretation of glibenclamide’s benefits and risks.

## Data Availability

The original contributions presented in the study are included in the article/supplementary material, further inquiries can be directed to the corresponding author.
